# Histopathological Study of Skin Adnexal Tumours—Institutional Study in South India

**DOI:** 10.1155/2014/543756

**Published:** 2014-02-05

**Authors:** Ankit Sharma, Deepak G. Paricharak, Jitendra Singh Nigam, Shivani Rewri, Priyanka Bhatia Soni, Anita Omhare, Preethi Sekar

**Affiliations:** ^1^Department of Pathology, D.D.U. Hospital, Hari Nagar, New Delhi 110066, India; ^2^Department of Pathology, Dr. D. Y. Patil Medical College, Kolhapur, Maharashtra 416006, India

## Abstract

*Objective*. The aim of this study was correlation of skin adnexal tumors with age, sex, and location and determining its incidence in the Department of Pathology at Dr. D. Y. Patil Medical College and Hospital, Kolhapur, Maharashtra. *Material and Methods*. 56 cases were included in this study from Jan 2004 to June 2010 with respect to incidence of adnexal tumors, age, and sex distribution. All slides were stained with haematoxylin and eosin and then findings were corroborated with special stains like PAS and reticulin wherever required. *Results*. 80.36% (45/56) were benign and 19.64% (11/56) were malignant adnexal tumors. The sweat gland tumors constituted the largest group (42.86% 24/56) cases followed by the hair follicle tumors (35.71%, 20/56) of cases and sebaceous gland tumors (21.43%, 12/56) cases. Overall male : female ratio was 1.07 : 1. The commonest age group was 51–60 years and the commonest affected body part was head and neck region (64.28%, 36/56) followed by trunk (14.28%, 8/56). Clear cell hidradenoma and pilomatricoma were commonest benign tumors and sebaceous carcinoma was the only malignant tumor seen. *Conclusion*. The incidence of benign skin adnexal tumors was more as compared to the malignant tumors. Malignant tumors were seen in older age group, usually over 50 years of age.

## 1. Introduction

Skin adnexal tumors (SATs) are those neoplasms that differentiate toward or arise from pilosebaceous unit, eccrine sweat glands or apocrine sweat glands, and these tumors are classified into four groups that exhibit histologic features analogous to hair follicles, sebaceous glands, and eccrine glands [1]. These tumors are derived from multipotential undifferentiated cells present within the epidermis or its appendageal structures and the histologic features of a tumor are related to the activation of molecular pathways responsible for forming the mature adnexal structure [1].

Most of the benign SATs present as asymptomatic papules or nodules and often difficult to diagnose clinically however anatomic location, number and distribution of lesions provide important clue, No change required [2]. They are however confirmed by histopathology, and immunohistochemistry may helps in confirmation of the diagnosis [3]. This study was therefore undertaken to analyze adnexal tumors of the skin for their morphological, clinical, and histological features and to group them using the International Classification of World Health Organization (2006).

## 2. Material and Methods

The present study includes the cases from Jan 2004 to June 2010. 56 cases were included in this study, which were reported by the Histopathology Sections of the Department of pathology at Dr. D. Y. Patil Medical College and Hospital, Kolhapur, Maharashtra. The clinicopathological data was taken from the record office for the given period. The histopathological examination was done on formalin fixed tissues and paraffin embedded blocks were made. Haematoxylin and Eosin stained sections were examined and few special stains like PAS and reticulin were performed wherever required. However in our laboratory setup, the facility for histochemical staining for enzymes like alkaline phosphatase, phosphorylase, succinic dehydrogenase, indoxyl esterase, and acid phosphatase was not available; they were not performed.

## 3. Results

In the present study, benign adnexal tumors constituted 80.36% (45/56) cases and malignant adnexal tumors constituted 19.64% (11/56) cases. The sweat gland tumors constituted the largest group involving 42.86% (24/56) cases followed by the hair follicle tumors 37.71% (20/56) cases followed by sebaceous gland tumors 21.43% (12/56) cases (Table 1). The male: female ratio was 1.07 : 1. Tumors were observed in all age groups ranging from 10 to 88 years. However, the highest incidence was observed in the age group of 51–60 years (26.78%, 15/56) followed by age groups of 31–40 (16.07%, 9/56) and 41–50 (14.28%, 8/56) years, respectively. The head and neck region was the most common site affected (64.28%, 36/56) followed by trunk (14.28%, 8/56) and then upper limb (12.5%, 7/56). In head and neck region 37.5% (21/56) cases were located on the face followed by scalp in 21.43% (12/56) cases. The neck region was least affected 5.36% (3/56) (Table 2).

**Figure 1:**
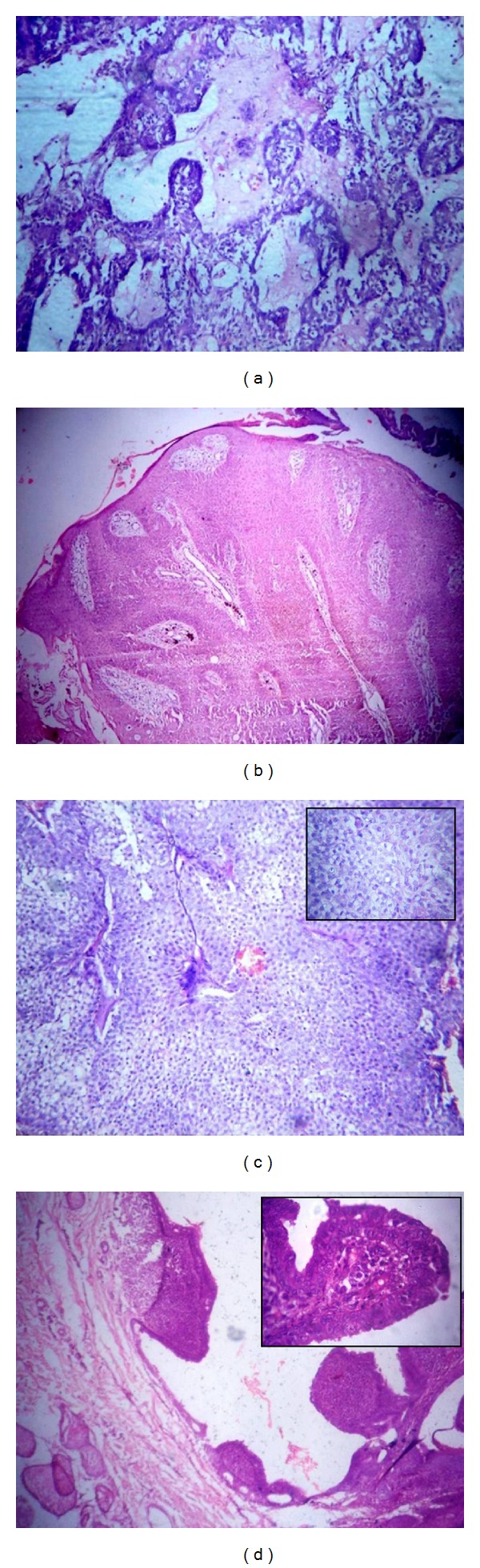
(a) Chondroid syringoma: tubular or ductular proliferation admixed with a chondroid stromal matrix. (H&E ×100). (b) Eccrine poroma: well-circumscribed tumor arising from lower portion of the epidermis extends downwards into dermis as cords and broad columns of uniform basaloid cells (H&E ×100). (c) Hidradenoma: Well-circumscribed nonencapsulated multilobulated tumor lobules with varying size and shape (H&E ×100), consisting of polyhedral cells with round nuclei with slightly basophilic cytoplasm and round cells with clear cytoplasm with small dark nuclei (Inset H&E ×400). (d) Syringocystadenoma papilliferum: epidermis with papillary projections extend into the lumina of the cystic invaginations. (H&E ×100) and the luminal row of high columnar cells with oval nuclei and faintly eosinophilic cytoplasm (Inset H&E ×400).

**Figure 2:**
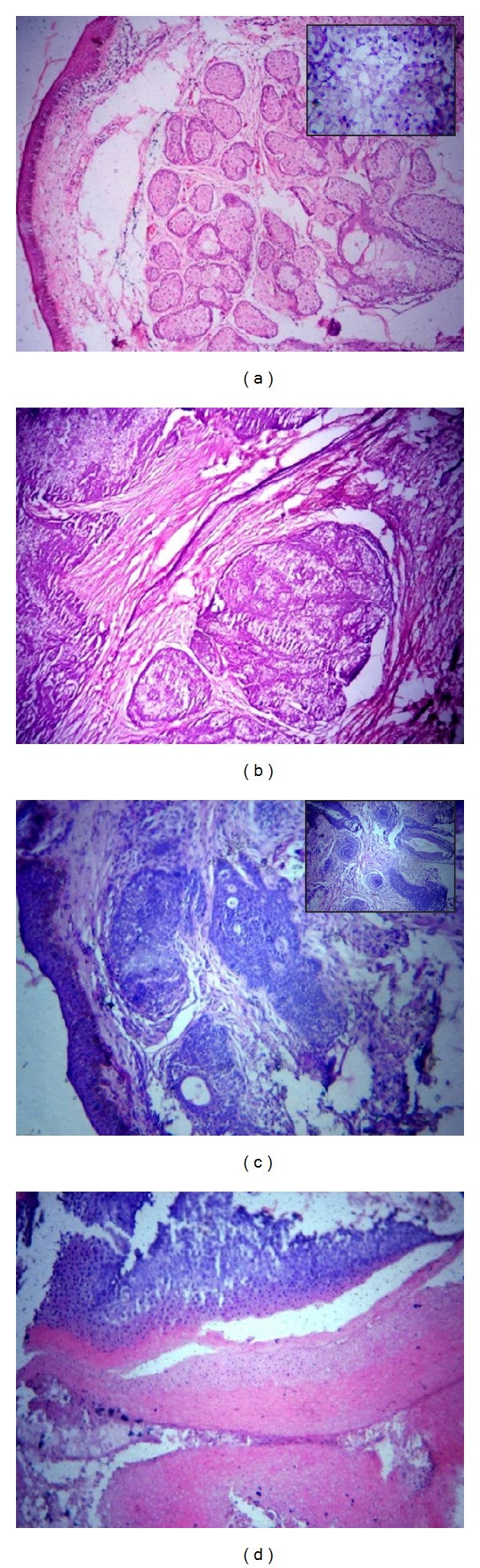
(a) Sebaceous adenoma: well-circumscribed tumor composed of sebaceous lobules of varying sizes and shapes (H&E ×100). Undifferentiated basaloid cells at the periphery while mature sebaceous cells were seen towards their center (Inset H&E ×400). (b) Sebaceous carcinoma: lobular arrangements of cells consist of small undifferentiated eosinophilic cells and large cells with clear to foamy cytoplasm. (H&E ×100). (c) Trichofolliculoma: dermis showing multiple cysts filled with keratin and lined by squamous epithelium (H&E ×100) and multiple cysts filled with keratin and lined by squamous epithelium. (Inset H&E ×100). (d) Pilomatricoma: Eosinophilic shadow cells predominantly and few basophilic cells with keratinization. (H&E ×100).

The sweat glands tumors are comprised of chondroid syringoma, eccrine poroma, nodular clear cell hidradenoma, apocrine hidrocystoma, and syringocystadenoma papilliferum (Figure 1). Sebaceous glands tumors are comprised of sebaceous adenoma and sebaceous carcinoma. The hair follicle tumors are comprised of trichoepithelioma, trichofolliculoma (sebaceous), pilomatricoma, and proliferating trichilemmal cyst (Figure 2). Amongst the benign tumors; Clear cell hidradenoma and Pilomatricoma were the most common tumors representing 12 (21.43%) cases each, clear cell hidradenoma was observed in age ranging from 10 to 70 years. Most of the patients were above 40 years of age and female preponderance. pilomatricoma showed peak incidence between 11 and 40 years of age and was more common in males. 6 (10.71%). cases of eccrine poroma were seen with age group ranged from 11 to 60 years and were more common in females. Chondroid syringoma came next, constituting 3 (5.36%) cases, out of which 2 (3.57%) were observed in patients above 30 years of age (Table 3). Amongst the malignant tumors sebaceous carcinoma was the only malignant tumor observed and constituted 19.64% (11/56). Most of them were meibomian gland carcinomas occurring in people above 50 years of age with maximum number of cases, that is, 4 observed in females above 70 years of age.

## 4. Discussion

Incidence of benign tumors is more as compared to malignant cases. In present study 80.36% (45/56) tumors were benign and 19.64% (11/56) tumors were malignant which was also seen in studies of Radhika et al. [4], Reddy et al. [5], and Samaila [6] who reported 77.14%, 69.41%, and 88.5% benign and 29.63%, 30.59%, and 11.5% malignant lesions, respectively. Nair [7] observed that sweat glands tumors are the commonest followed by hair follicle tumors and then sebaceous glands tumors. The present study also shows similar results. However, Radhika et al. [4] and Samalia [6] observed that sweat glands tumors are the commonest SATs followed by sebaceous glands tumors followed by tumors of hair follicle. Male : female ratio as observed by Nair [7] and Saha et al. [8] was 1 : 2.3 and 1 : 1.88, respectively. Radhika et al. also observed that majority of the patients are in the third decade and females outnumbered males [4]; however, present study showed male : female ratio as 1.07 : 1. Saha et al. [8] observed the mean age of onset of SATs was 24.15 ± 8.44. Nair [7] observed the commonest age group of presentation was 11–20 years; however, in the present study, commonest age group was 51–60 years followed by 31–40 years. Samalia [6] observed that 46% of lesions were located in head and neck region which was also seen in our study. Song et al. observed that pilomatricoma was the most common benign tumor followed by dermoid cyst followed by steatocystoma multiplex, syringoma, and trichilemmal cyst [9]. Radhika et al. observed that the most common benign tumor is nodular hidradenoma followed by sebaceous naevus [4]. In present study, most common tumors were clear cell hidradenoma and pilomatricoma followed by eccrine poroma.

## 5. Conclusion

In Indian population, an overall incidence of skin adnexal tumors is very low. The incidence of benign skin adnexal tumors is more as compared to the malignant ones. Most of the malignant tumors occur in older age group usually over 50 years of age. However benign tumors show a wide age variation. Skin adnexal tumors can occur anywhere in the body; however head and neck region constitutes the most common site. Majority of the tumors can be classified into different subgroups on the basis of light microscopy alone. Skin adnexal tumors showing sweat gland differentiation are seen more frequently. In our institutional study, clear cell hidradenoma is the commonest tumor with sweat gland differentiation, while pilomatricoma is the most common type of hair follicle tumor. Amongst the tumors with sebaceous differentiation, sebaceous carcinoma (meibomian carcinoma) is commonest.

## Figures and Tables

**Table 1:** Adnexal tumors according to the direction of differentiation.

Sr. no.	Direction of differentiation	No. of cases	Percentage incidence (%)
1	Sweat gland tumors	24	42.86
2	Sebaceous gland tumors	12	21.43
3	Hair follicle tumors	20	35.71

	Total	56	100.00

**Table 2:** The site and sex distribution of observed adnexal tumors.

Sr. no.	Site of tumor	Male	Female	Total	Percentage incidence (%)
	Head and neck				
1	Scalp	5	7	12	21.43
	Face	9	12	21	37.5
	Neck	2	1	3	5.36
2	Trunk	6	2	8	14.29
3	Upper limb	5	2	7	12.5
4	Lower limb	1	3	4	7.14
5	Not specified	1	—	1	1.78

	Total	29	27	56	100.00

**Table 3:** Age incidence of individual adnexal tumors observed in the present study.

Sr. no.	Tumors	Age groups (in years)							
		1–10	11–20	21–30	31–40	41–50	51–60	61–70	>70
	Sweat gland tumors								
1	Chondroid syringoma			1	1		1		
2	Eccrine poroma		1		1	2	2		
3	Nodular hidradenoma	2	2			4	3	1	
4	Apocrine hidrocystoma							1	
5	Syringocystadenoma papilliferum		1	1					

	Sebaceous gland tumors								
6	Sebaceous adenoma						1		
7	Sebaceous carcinoma					1	4	2	4

	Hair follicle tumors								
8	Trichoepithelioma			1	1				
9	Trichofolliculoma (Sebaceous)			1	1				
10	Pilomatricoma	1	2	2	4	1	1	1	
11	Proliferating trichilemmal cyst				1		3		

	Total	3	6	6	9	8	15	5	4
